# Producer Perceptions Toward Prevention and Control of Lameness in Dairy Cows in Alberta Canada: A Thematic Analysis

**DOI:** 10.3389/fvets.2022.812710

**Published:** 2022-02-08

**Authors:** Marlena Knauss, Cindy L. Adams, Karin Orsel

**Affiliations:** ^1^Department of Production Animal Health, Faculty of Veterinary Medicine, University of Calgary, Calgary, AB, Canada; ^2^Department of Veterinary Clinical and Diagnostic Sciences, Faculty of Veterinary Medicine, University of Calgary, Calgary, AB, Canada

**Keywords:** qualitative research, in-depth interview, thematic analysis, producer perception, veterinary communication, lameness, dairy cow

## Abstract

Lameness in dairy cattle poses both an animal welfare and economic threat to dairy farms. Although the Canadian dairy industry has identified lameness as the most important health issue, lameness prevalence in the province of Alberta has not decreased over the last decade. Factors related to lameness have been reported, but the prevalence remains high. Therefore, this study was conducted to investigate dairy producers' perceptions on lameness and how these perceptions influence lameness prevalence in their cows. Qualitative interviews with open-ended questions were conducted with nine dairy producers in Alberta, Canada presenting farms with a wide variety of lameness prevalence. Thematic analysis of these interviews revealed five major themes, as well as five distinct types of producers regarding their perceptions. All nine producers mentioned similar challenges with lameness prevention and control. Identifying lameness, taking action, delays in achieving success, various approaches to prevention and control strategies, and differences between farms were the challenges encountered. However, producers' attitudes when dealing with these challenges varied. We concluded that understanding producers' perceptions is essential as no “one size fits all”, when advising them regarding how to address lameness, as guidance and support will be most successful when it is aligned with their viewpoint.

## Introduction

Canadian dairy producers identified lameness as the most important dairy cattle health issue (1). However, lameness prevalence has remained ~20% over the last decade in Alberta, Canada (2, 3). The negative effects of lameness include decreases in farm profitability (4), cow longevity (5), productivity (6), and reproductive performance (7). Lameness is also an animal welfare issue (8), as it is a clinical sign of pain resulting in an altered gait (9). These facts raise the question as to why the prevalence of lameness on most dairy farms commonly surpasses the Canadian Dairy Code of Practice's recommendation of 10% (10). To investigate this issue, two quantitative studies were conducted at the University of Calgary, Faculty of Veterinary Medicine (UCVM). Risk factors for lameness were evaluated in 2011 and 2012 (2), followed by a 2018 study to elucidate associations between lameness and these risk factors, using an on-farm risk assessment questionnaire (RAQ) on 65 dairy farms in Alberta, Canada (3). Lameness prevalence was determined by assessing gait in videos. The lameness RAQ contained questions pertaining to lameness risk factors. In the 2018 study, it was hypothesized that farms with a high score on the risk assessment also have more risk factors present for lameness. Unexpectedly, the correlation between risk factors and lameness prevalence was only modest (3), suggesting a complex association between identified risk factors and the presence of lameness. However, human factors influencing on-farm decisions and management were not included in this RAQ.

Despite new knowledge on the pathophysiology of and risk factors for lameness (11–13), plus an increased focus on communicating herd health issues with dairy producers (14), lameness has not been reduced. Although quantitative questionnaires have been used to study barriers in lameness prevention and control (15, 16), this approach does not necessarily capture the complexity of drivers of behavioral change in humans. Understanding the motivators behind actions is crucial to develop strategies for working on solutions for complex health issues like lameness (17). To better understand the perspectives of producers that are difficult to elicit through standardized survey methods, qualitative research is becoming increasingly important in dairy science to gain a thorough understanding of dairy producers' perceptions, attitudes, and beliefs when implementing prevention and control strategies (18). In qualitative research, theories such as the Health Belief Model (19) and the Theory of Planned Behavior (20) can be used to better understand peoples' behavior, and shed light on motivators and drivers that help to determine peoples' actions.

The objectives of this study were to garner a thorough understanding of the dairy producers' perceptions, attitudes, and beliefs about lameness prevention and control, along with a better understanding about challenges in addressing lameness and opportunities for support in managing lameness.

## Materials and Methods

The study protocol and interview guide were approved by the University of Calgary Research Ethics Board (REB17-1522).

### Selection of Producers

**Figure 1:**
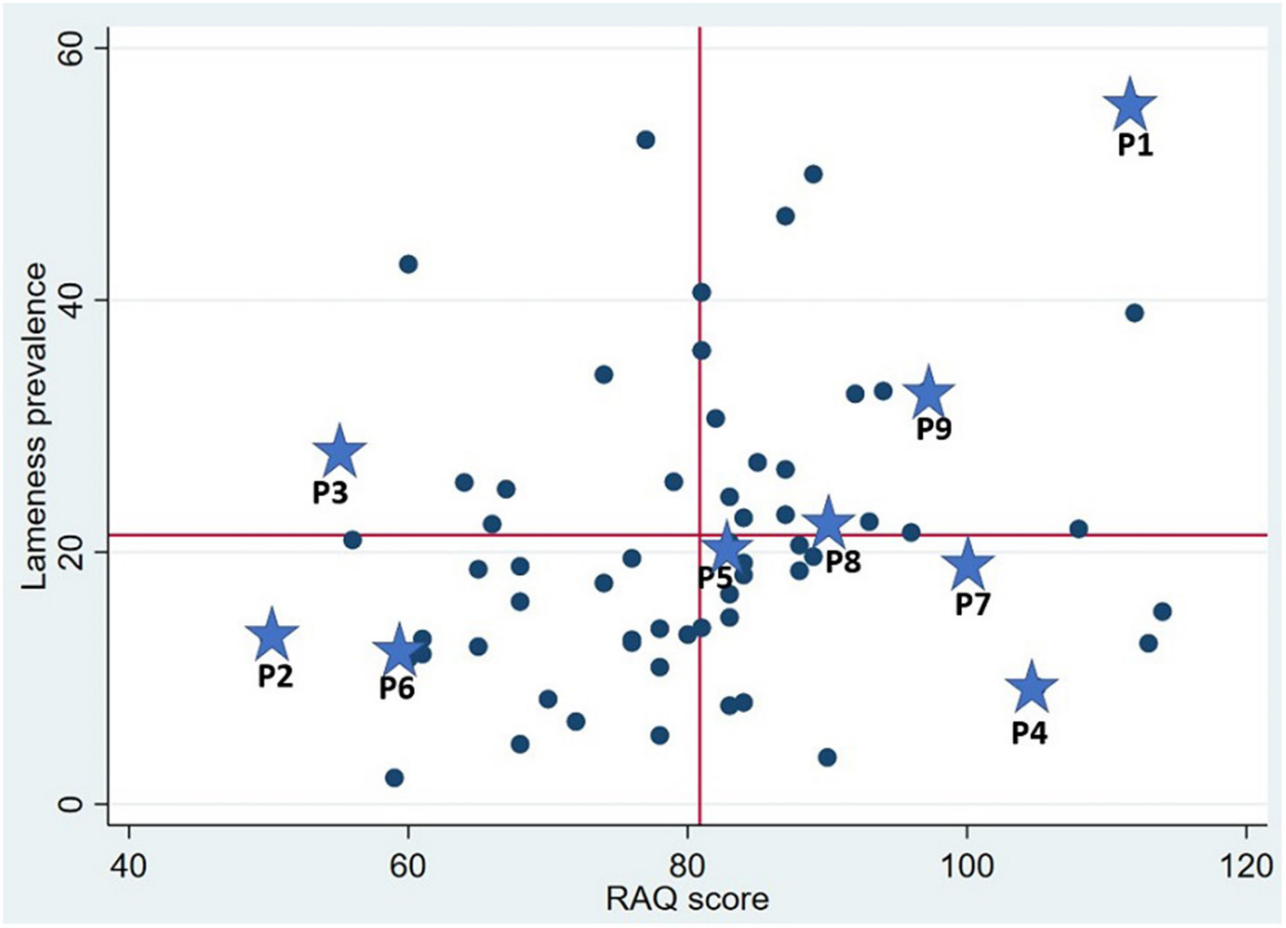
Scatterplot to identify groups of participants. X-Axis, Risk assessment score with gridline at the mean score. Y-Axis, Lameness prevalence with gridline at the mean prevalence. Stars in combination with P 1–9 mark the participants.

Dairy producers participating in the 2018 UCVM lameness study (3) comprised the sample for the current study. To capture a variety of perspectives, purposive sampling was used for recruiting; based on lameness prevalence and RAQ results, a scatterplot was created (Figure 1). This scatterplot was used to group farms into the following categories: (1) High lameness prevalence/High risk for lameness (*n* = 3); (2) Low lameness prevalence/Low risk for lameness (*n* = 2); (3) High lameness prevalence/Low risk for lameness (*n* = 2); (4) Low lameness prevalence/High risk for lameness (*n* = 3); these were designated the 4 “extremes;” whereas the final category, (5) Medium lameness prevalence/Medium risk assessment score (*n* = 55), was designated the “core.”

**Figure 2:**
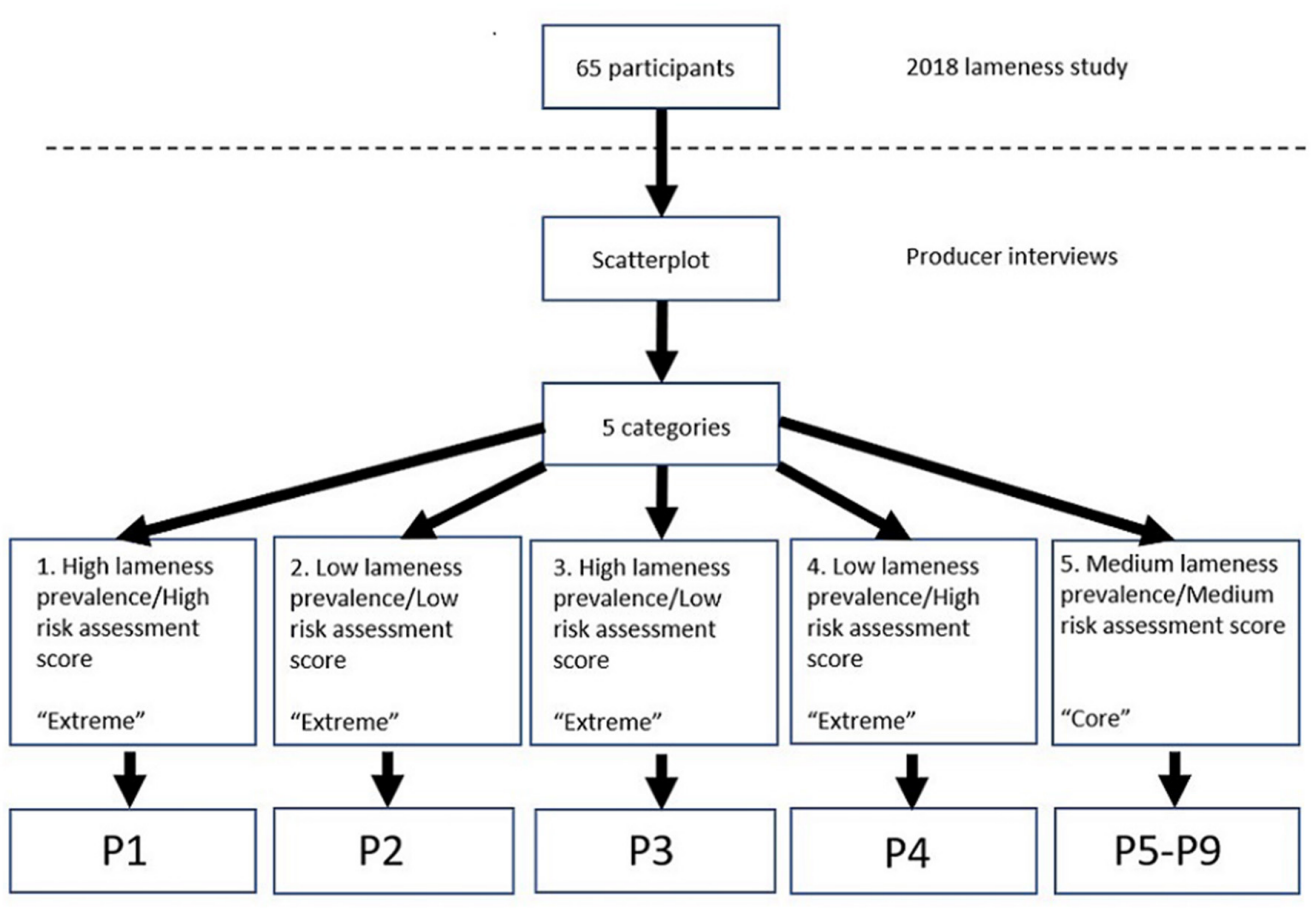
Flowchart to represent the selection and categories of participants.

All producers who were identified as extremes for lameness and/or risk (*n* = 10) were contacted. One producer from each extreme (*n* = 4) was willing to be interviewed. To increase the number of participants, randomly selected producers from the core category were contacted. Thematic saturation was achieved after a total number of nine interviews (extremes *n* = 4, core *n* = 5). A summary of the selection of the participants is shown in Figure 2.

### Data Collection

A successful interviewer is knowledgeable in the field to understand the lingo and build rapport with the interviewees. The interviewer, first author MK, is a veterinarian and graduate student. MK has 12 years of experience in the dairy industry, with 1 year focussing specifically on lameness. During her graduate education, she received training in qualitative data collection and analysis. The interview language was English; however, MK's first language is German. The interviewees were aware of MKs professional veterinary background; however, MK was unfamiliar to the producers.

The number of participants in the study was determined by a desire to conduct a deep level of analysis. For deep analysis, 6–12 participants were recommended (21, 22). To ensure validity, reliability, and generalizability/transferability pertaining to a qualitative research theme definitions of these terms by Finfgeld-Connett (23) and Leung (21) were studied. Validity refers to the use of appropriate study design, where reliability refers to consistency in data analysis. Generalizability is not a desired asset in qualitative research, instead transferability is important. Transferability pertains to transferring a theoretical framework from one context to another (23, 24) Existing guidelines for data analysis were followed, which are described in more detail in section “Thematic Analysis” (24). Saturation was used to determine the end of the interview series (25), whereby no new themes were identified after interview number 6. The three remaining interviews were used for confirmation purposes.

Interviews were conducted between June and August 2019. The interview process was based on inductive inquiry (26). Data collection was facilitated using a semi-structured open-ended interview guide, with questions about demographics, attitudes and beliefs, knowledge, and so-called external stimuli or information sources (Supplementary Material - Interview guideline). The semi-structured interview guideline was developed by the authors guided by semi-structured interview guidelines in published papers in the field (16, 27). The producers were encouraged to guide the conversation; however, follow-up questions were asked to gather more information on specific topics mentioned by the producers. Producers were asked explicitly about the impact of being audio-recorded at the end of the interview. The interview guide designed and evaluated by the authors was pilot tested on an Albertan dairy producer not included in the 2018 UCVM lameness study for face validity and an estimate of time to complete. All interviews were done on farm; two were conducted in the kitchen of the farmhouse and seven were conducted in the barn office. The interviewer did not inspect the facility nor the cows, as that was outside the scope of this project. Field notes were written following visits to the farms where descriptions of the farm and producers were noted. The field notes included the overall impression of the farm outside the facility itself (e.g., overall impression of tidiness and cleanliness) and the impression of the producer (e.g., was he willing or hesitant to talk). Observations deemed noteworthy or that stood out were captured. Field notes were used as reference throughout the analysis.

All interviews were conducted on separate days and audio-recorded with two audio-recorders (Sony ICD-UX560, Sony Corporation, Tokyo, Japan as the primary, with a Philips DVT1150, Speech Processing Solutions GmbH, Vienna, Austria as backup). Audio-recordings were transcribed verbatim by MK, on days between the interviews, using specialized transcription software (Express Scribe Transcription Software Pro v 8.06, NCH Software, Greenwood Village, CO, USA). Transcripts were typed in Microsoft Word (Microsoft Corporation, Redmond, WA, USA). Transcribed interviews served to inform the inclusion of further probing questions during subsequent interviews.

### Data Analysis

#### Thematic Analysis

Following Braun and Clarke (24), thematic analysis of the interviews consisted of six phases:

1) Familiarization with the data
  The process of familiarizing with the data commenced the reflective process that was required for ongoing interpretation of the data collected
2) Generation of initial codes
3) Search for themes
4) Reviewing themes including peer review of transcripts group consensus confirmation of accuracy by producers
5) Defining and naming themes and
6) Producing the report

Transcription and repeated listening to recordings facilitated complete familiarization with the data. The analysis of the content started with transcription and multiple readings of the transcripts. Initial ideas, comparisons and connections were noted.

To generate initial codes, verbatim transcripts of the audio-recorded interviews were coded using coding software (NVivo, QSR International Pty Ltd. Version 10, 2012). Two coding cycles were used to analyze the transcripts following Saldana (28). For the first step, both *in-vivo* and holistic coding were used. *In-vivo* coding focuses on the participant's voice and uses actual phrases of the record as code. Holistic coding is an approach to cover the whole meaning of statements in the interview (28). For the second step, focused coding was used to gather the most frequent and significant codes from the first coding cycle (28). After the second coding cycle, codes were analyzed for themes that occurred in the interviews as recurring patterns as part of the third step (28). Because qualitative analysis is an iterative process, the developed concepts were refined over time.

Nominal group technique (NGT), which is a formal consensus development method (29) was employed to ensure reliability of the codes. NGT aims to bring reliable qualitative information by consensus of a group of experts on a specific topic, where the consensus is obtained in discussions in face-to-face meetings (29). For this purpose, the authors met regularly to discuss the codes, emerging themes within these codes and their deeper meaning. The authors met 12 times, with each meeting lasting up to 1.5 h. The first author guided the discussion by presenting important topics and how they related to each other. The co-authors encouraged discussion, to ensure reliability of interpretation of certain themes. Reliability in qualitative research refers to consistency in data analysis (21), which was obtained through NGT. These meetings resulted in conceptualization of the deeper meanings of the interviews. During group meetings, themes were defined, and the name of these themes were developed to ensure that the meaning of the themes was captured in a clear manner. Finally, during the analysis phase, the co-authors met and discussed all parts of the report to ensure thorough analysis of the data and the validity of the analysis.

### Member Checking-Participant Validation

Results of the study were returned to all the producers as a newsletter with e-mail or mail to check for accuracy and resonance with their experiences. Moreover, the findings were presented on a poster at the Western Canadian Dairy Seminar (WCDS) in Red Deer in 2020. Four of the nine participants reviewed the findings and those four producers expressed their agreement with the authors results. Two of the producers responded to the e-mail and two were at the WCDS. The other five did not respond.

## Results

When asked how they felt about being audio-taped, all the producers indicated that being recorded did not impact the quality of the interview. The interviews lasted from 26 to 87 min. In the results, the producers (P) were identified as P1–P9: P1 belonged to category 1, P2 belonged to category 2, P3 belonged to category 3, P4 to category 4, and P5–P9 belonged to category 5 (Figure 2).

### Demographics

All the producers were male. The actual age of the producers was not obtained. We estimated that they were between 30 and 70 years old, because during the interviews they narrated when they started in the dairy industry and how long they are in the dairy industry now. All the producers assumed the role of the dairy managers on their farm. All grew up on a dairy farm in either Europe or Canada and had a life-long connection with agriculture. Herd sizes ranged from 60 to 300 dairy cows. Levels of education spanned from self-taught (learning by doing), a high school degree, agricultural college, or agricultural university.

### Thematic Analysis

Five major themes were identified during the analysis. These themes reflected producers' perception of lameness prevention and control:

Theme 1. Identifying lameness poses a problem.
  Subtheme 1.1. Barn blind.
  Subtheme 1.2. Lack of ability to detect lameness.
Theme 2. Responsibility for lameness prevention and control.
Theme 3 Success is not immediate and hard to measure.
Theme 4. Decision making for and against lameness prevention and control.
Theme 5. One size does not fit all.

#### Theme 1. Identifying Lameness Poses a Problem

##### Subtheme 1.1. Barn Blind

For the producers in this study, it was generally a challenge to determine that a cow was lame; and this was referred to as “barn blind.” Producers explained that differences in locomotion scoring among their peers or others (e.g., veterinarians, researchers, and farm advisors) might include not agreeing on whether a cow is lame or sound due to the subjectivity of lameness scoring. Producers also described themselves as “barn blind,” which means that they do not see the problems on their farms anymore, due to consistently being in the same environment.

All the producers were disappointed by their lameness scoring in the 2018 UCVM lameness study and they described that study as an “eye-opener” to the true lameness prevalence on their farms.

A reason for the high lameness prevalence was described as: “Well, I think the first barrier is, is the realization or the identifying lameness. […] If you don't think its lame, you are not going to fix it.” and: “Like sometimes as an owner, you are just blind. Like you don't see it anymore on your own farm.”

##### Subtheme 1.2. Lack of Ability to Detect Lameness

Producers reported that lameness is often hard to diagnose and that lame cows are often not recognized until they are severely lame. Only P2 stated that he used locomotion scoring to determine lame cows. Producers remarked that they had some training on locomotion scoring as a part of a hoof trimming workshop, but not as a stand-alone workshop. Not all producers were interested in a scoring-focused workshop, due to time constraints. A valued source of learning identified by the producers was observing the hoof trimmer and asking questions.

#### Theme 2. Responsibility for Lameness Prevention and Control

All producers stated that they believe that there is not a single farm without some degree of lameness. A lameness prevalence of zero does not seem realistic to producers; however, producers stated that they were working on keeping lameness under control. All producers, except for P1, agreed that taking actions to prevent and control lameness was their responsibility and they were capable to do so: “No, it has to be the producer, the producer has to. I mean, it has to start, and it has to end with us. Like, we're ultimately responsible for, for the cows that we're, we're looking after.” In contrast, P1 envisioned himself in a position where the cause of lameness determined whether he could address lameness prevention and control. Furthermore, the importance of lameness varied among producers. Although, none of the producers described lameness as their most important issue, it was described as very important, along with other health problems like mastitis and fertility.

#### Theme 3. Success Is Not Immediate and Hard to Measure

Whether an implemented lameness prevention strategy was successful is not apparent immediate, which was identified as a challenge by the producers. Due to the delay, producers were likely to forget what they implemented months ago and fail to associate the positive outcome with that intervention. This was revealed as a frustrating aspect of lameness prevention and control. Moreover, the effect of success in addressing lameness is hard to measure for producers. However, they think that failure is easier to measure, because not being able to have lameness under control means losses in both productivity and money: “Well, some of the effects they do not show up right away. […] It's easier to measure when it doesn't work.”

#### Theme 4. Decision Making for and Against Lameness Prevention and Control

A big driver in decision-making regarding whether to implement a certain prevention and control strategy, is the perceived financial situation of the farm, e.g., whether the producer is able to invest money in lameness prevention and control. To take the financial risk when investing, producers had various expectations about strategies: P1 and P5 wanted to be convinced that it worked, without being able to specify how they could be convinced, whereas P2 wanted to see the research behind the strategies to decide whether it was worth the investment. Furthermore, P3 and P9 were only willing to implement simple strategies, without being able to specify what simple meant to them in this context. Both P4 and P7 wanted proof that it worked on other farms before they were willing to implement these strategies. Regardless, the reason for investing in prevention and control strategies was the same for eight of the nine producers; they said that it is cheaper to prevent lameness than to treat it.

#### Theme 5. One Size Does Not Fit All

Producers identified multiple components as part of a prevention and control program as a challenge, as no “one size fits all.”

Producers understand that certain strategies do not have the same effect on every farm: “You know, for some places, it works good in one way; in other places, it works good in different ways.” Because there is no “one size fits all” approach in lameness prevention and control, producers struggle to prioritize what is best. Dairy farmers struggle, because they face the inability to filter the overwhelming load of information on lameness prevention and control strategies to develop feasible strategies for their farm: “Well there is a lot of information; it's almost an information overload.” Producers admitted talking often to other producers, however, it was difficult for them to critically appraise whether the strategies implemented by their fellow producers would have a similar effect on their farm. To deal with that issue, producers sought one-on-one expert advice, because they acknowledged that they needed an outside opinion to understand the main issues on their farm. Producers believe that a team approach to advice could deliver some solutions to lameness issues. During interviews, a team approach was defined by the producers as a consultation where all important lameness advisors (e.g., veterinarian, hoof trimmer, and nutritionist) were present and perhaps even other producers who were successful in addressing lameness on their own farm. However, pertaining to their satisfaction with their veterinarians' advice on lameness, producers did not agree. Some felt that their veterinarian explained lameness prevention and control well and they learned a lot. Others expressed a lack of understanding of their veterinarian's information dissemination. Others did not receive any direction from their veterinarian.

To emphasize that importance of one-on-one advice tailored to the producer and their situation, five characteristics stood out among the participants and are elucidated in the following.

#### The Overwhelmed Producer

Although, P1 was concerned about the welfare of his cows, he was not successful in addressing lameness. During the interview, he seemed overwhelmed with the workload on his farm, mostly managing the farm on his own with limited help. He was reflecting on the fact that he was older and that he did not have sufficient labor to complete all the work. He stated, “I wish I could do more,” when talking about how satisfied he was with addressing lameness. Moreover, he said, being in the dairy industry for a long time, he had acquired much information on lameness from peers and advice along the way. In conclusion, P1 tried to address all his farm responsibilities, but his workload was overwhelming.

#### The Eager Producer

P2 was the only producer who stated that animal welfare was of utmost importance on his farm and that the economic benefit only came second. He identified lameness control and prevention as the sole responsibility of producers and put effort in it. As a result, he was classified in category 2, because he was successful in managing lameness. He actively searched and implemented new knowledge to use on his farm. Through reflection on evidence presented on a new control strategy, he identified suitability for his facility set-up and management. He would intervene immediately in response to a new lameness case and felt the benefits outweighed the additional workload.

P3 shared his responsibilities with his wife and others (their children, a hired herd manager and milkers). This producer obviously took pride in his herd and his wife provided additional care for every cow. Despite having a 300-cow operation, his wife knew each cow as an individual. When being asked why he thought he belonged to category 3, he stated, that he just moved in the new barn before the evaluation in 2018. The producer guessed that the lameness prevalence should be lower now compared to a year ago, because cows should have adjusted to the new environment. Much thought had been put in planning the new barn, as the producer gathered information on barn systems, and he observed his relatives' success with a similar barn. Moreover, this producer also tried to keep himself updated on new information regarding lameness prevention and control. In summary, this producer was not only keen on keeping his cows healthy, but also on having a successful family enterprise.

#### The Collaborative Producer

P4's facility was not ideal, according to the RAQ score from 2018; however, lameness was also a focus and this producer attempted to immediately address new lameness cases. He also stated that they would have enough workforce on farm to address lameness cases immediately. Moreover, the work force consulted with each other when planning to implement prevention and control strategies. When convinced of a rational strategy, it was implemented. In the interview, P4 said that communication among farm personnel was the most important method to successfully address lameness and that they had achieved their goal of minimizing lameness.

P6 also had additional labor available. Despite only two people being responsible for the daily dairy work, many people consulted with each other when decisions were made regarding implementing lameness prevention and control strategies. P6 was eager to collect information on prevention and control strategies and he actively contacted researchers and other consultants for one-on-one advice. Knowing that his facility and lameness prevalence on his farm were only average, he strived to convince all decision-makers on farm to invest in new technologies to improve not just lameness, but also overall cow health.

#### The Transitioning Producer

P7 was trying to transfer responsibilities to his son, who was still in school. He acknowledged that this can only happen step by step, but he was no longer willing to further educate himself on lameness. In his opinion, his son should participate in all educational events when it was time for him to take over the farm and he saw no value in investing more effort himself.

The transition, however, can also occur in the other direction. P5 and P9 were managers of their dairy; however, for both, their parents still had a huge influence on decision-making. Both producers conveyed their ideas on how to address lameness and strategies to implement. Notwithstanding, they made it clear that it was not their sole decision and that they needed to convince their parents before making changes.

#### The Convenient Producer

P8 only had irregular, part-time help on his farm. Therefore, his focus was on prevention and control strategies that were convenient. This producer honestly admitted that the reason why he was negligent with his foot bathing protocol was that it was inconvenient. He reasoned that he would be more rigid on footbaths if it was more convenient, but he lacked the funds to make improvements. Although, this producer had identified his weakness, he did not see himself in a position to change.

## Discussion

All producers mentioned the same challenges with lameness prevention and control; however, there were distinct differences among producers in their attitudes toward dealing with the challenges. These differences pertained to the five characteristics of producers that were identified in this study. However, more characteristics might have been identified with a wider sample, therefore, we do not conclude that the characteristics are exhaustive. Where the overwhelmed producer did not actively take action in lameness prevention and control, the eager producer investigated every single opportunity to thrive. Like the overwhelmed producer, the transitioning producers were also hesitant to take action. The collaborative producers took the opportunity to discuss strategies with their peers, which results in various input and perspectives. This is in line with an Australian study, as it suggested that discussions within the family might have a positive impact (30). The convenient producer is not facing the challenge but waiting for somebody to take on the challenge for him. Overall, only the eager and collaborative producers were facing the lameness challenge and trying to improve, where the other producers were waiting for someone else to take on that challenge for them. It is likely that the eager and collaborative producers did face the challenges because they have their peers where they sought advice. For the other producers age and experience might be limiting factors. Where the overwhelmed producer was the oldest participant, who also was in the industry the longest, it seemed as he wished to have someone who will take over. For the transitioning producer they either wanted to hand over to the younger generation, but they had no experience, or they were still patronized by their elders. The convenient producer was the youngest and might lack some experience because he is waiting for someone to guide him.

All the producers were disappointed about the lameness score obtained on their farm in the 2018 UCVM study, as they did not expect to score so poorly. Although not talking about a specific number of their scoring estimation, the phenomenon of underestimating the lameness prevalence on their own farm was in accordance with a previous study, where trained researchers reported the lameness prevalence to be 3.6 times higher than the producer (31). Producers generally described their inability to identify lame cows because the altered gait of lame cows had become the “new normal” gait to them, as they saw it daily, which was also described in studies by Leach et al. (15) and Cutler et al. (31). In social science, a shift of normality happens in other contexts too, as people can acquire different perceptions of what is normal (32). Producers who perceive a lame cow as normal do not see the need for taking action. If a producer is not aware of the problem there will be no changes; however, awareness can only be raised with constantly reminding the producer of the issue. Therefore, producer training on lameness detection or discussion with their veterinarians during herd health visits is beneficial for raising awareness.

All producers said that it is very likely for a dairy farm to have lame cows. However, they also stated that it is not the most important health issue on their farm and that they would take care of the most pressing health issues first. Producers identified mastitis and fertility problems as more important because it is more obvious to them. Moreover, producers described that these issues are easier to fix, and the success is easier to measure. With lameness prevention and control strategies the challenge is that the onset of success is delayed, and it is hard to measure if a strategy was worth the investment. According to the Health Belief Model, people need to believe in the susceptibility and severity of a disease or condition in order to act. Based on the analysis of the interviews the participants only believed in the susceptibility but underestimated severity of lameness (19). Producers stated that they believe that every farm has lameness issues to a certain degree, and it is very likely to have lame cows, however, lameness does not seem severe enough, because it is not directly associated with monetary losses. Because mastitis means an immediate drop in milk production and fertility problems result in having no calf, which result in monetary losses, these conditions are perceived as severe. An understanding how lameness indirectly affects milk production and fertility issues is important to raise awareness that lameness severely impacts the health condition of a cow.

Furthermore, there were differences in labor force availability that affected attitudes toward lameness prevention and control. When labor was restricted, the overwhelmed producer saw himself unable to invest time in lameness prevention and control, as the daily work routine dominated his time. Continuity of successful control strategies were discontinued due to employee turnover. On the contrary, the eager producer regarded lameness prevention and control as essential to the point that he was willing to put time aside to invest in the success of his control program. Skills and knowledge also contributed to his success with lameness prevention and control. P4 and P6 had ample labor and financial resources; these factors are components of the Theory of Planned Behavior, which described that even if people are willing to act, they need to have access to factors like time, money, skills and cooperation of others to be successful in performing the chosen behavior (20). Other producers said that they would be willing to implement a prevention and control strategy, but money was a restriction to moving forward, which is in accordance with an Australian study, where cost was identified a potential barrier (30). Other research suggests that the mindset of people and what they believe in is the main driver behind actions (19). To understand the main drivers behind these actions is crucial to move forward in taking actions in lameness prevention and control. Implementing strategies depends on the believes of the producers and understanding these is crucial to overcome the challenges in moving forward.

Producers stated during interviews that they intended to use a team approach, with professionals from various sectors collaborating to identify solutions. Producers also said that they enjoyed events where they met other producers and were able to exchange information and stories of success and failure. These events could be utilized for focus groups on lameness prevention and control. Focus groups were described as an effective way to discuss prevention and control strategies for diseases in order to bring producers together to share ideas (18). Moreover, social pressure may be a reason for producers to adopt prevention and control strategies for diseases (18). This combination of social pressure and perceived equality in discussions may be useful to motivate producers to address lameness.

Variations in perceptions among producers may account for why advisors in the dairy sector (e.g., veterinarians, hoof trimmers, and nutritionists) will be a challenge to address prevention and control. Based on the differences identified in this study, we recommend advisors must spend time inquiring into what each individual producer knows, and what they have tried. It is also important for advisors to find out what producers might be capable of based on their knowledge, the money they want to invest and time they can put into lameness prevention and control due to workload on farm or other factors. Gathering this important information is necessary in order to be more effective in addressing prevention and control strategies specific to individual farms and the farmers. Investigation into these perceptions and integration of them into the plan going forward is essential in motivating producers to take action (14, 17). Some producers need more information and direction on the farm, e.g., the overwhelmed producer, whereas others, for example, the eager producer, might require more advanced information to support their desire to mitigate disease. It seems to be crucial for advisors to be able to understand the challenges that the producer is dealing with regarding lameness.

Focusing on the producers' needs and ways to address them properly, including communication training in a veterinary curriculum has been successful (17, 33). Also, the clinical communication patterns of veterinary practitioners influenced producer compliance and adherence to their suggestions (14, 34). To the authors' knowledge, this field has much potential for further research, especially communication skills training for addressing lameness prevention and control and follow-up to determine if the skills taught are being implemented on the farm, along with impacts on animal health. This relates back to statements by the producers made, that themselves do not believe in a “one size fits all” approach, but more in an individual tailored approach. To gather relevant information about the producer's individual situation and what strategies bear the biggest potential on farm, communication skills training support advisors to be trained in gathering the most important information. With improved communication skills relevant challenges for the individual producer can be identified and proper communication enhances compliance (14, 34).

### Study Limitations

Participation in these interviews was voluntary and therefore only producers who were willing to share their opinion were interviewed. In these interviews, producers shared what they were willing to share, and were encouraged to lead the interview with guidance by the interviewer. The low number of participants is also a limitation. Although, five characteristics of producers were identified in the interviews, we cannot conclude that these characteristics are exhaustive. Consequently, findings are not extricable to the general population of dairy producers. Another limitation is the lacking reference to age. Therefore, no conclusions how age affects dairy producers' perceptions, attitudes and beliefs about lameness prevention and control were made.

## Conclusions

During the interviews, no single prevention or control strategy was identified as the most successful for all farms, as there is no “one size fits all” approach. Regardless, the challenges described were the same for each producer. Furthermore, producers differed in how they were addressing these challenges in lameness prevention and control and how their perception of lameness influenced their decisions and actions. Producers in the study desired tailored advice for their farm, and previous studies suggest that communicating based on individual needs enhances producer compliance. Therefore, it is of utmost importance for consultants to investigate and develop an understanding of the individual producer's attitudes, beliefs, and the perceived challenges to achieving success. There is also a call for the delivery of information by the advisors that is understandable and contextually relevant to the producer in order to enhance the success of lameness prevention and control strategies.

## Data Availability Statement

The original contributions presented in the study are included in the article/Supplementary Material, further inquiries can be directed to the corresponding author/s.

## Ethics Statement

The studies involving human participants were reviewed and approved by University of Calgary Research Ethics Board (REB17-1522). The patients/participants provided their written informed consent to participate in this study.

## Author Contributions

MK conducted the semi-structured interviews, transcribed the interviews, did initial coding of the data, conducted ongoing analysis with input from CA and KO, and prepared the manuscript with input from CA and KO. CA provided training for MK, co-supervised MK for this project, conducted ongoing analysis with MK and KO, and reviewed drafts of this manuscript for publication. KO supervised MK for this project, initiated this project, conducted ongoing analysis with MK and CA, and reviewed drafts of this manuscript for publication. All authors contributed to the article and approved the submitted version.

## Funding

This study was funded by Alberta Milk (Edmonton, Alberta, Canada), and the Industry and Market Development Fund, Alberta Agriculture and Forestry (Edmonton, Alberta,Canada).

## Conflict of Interest

The authors declare that the research was conducted in the absence of any commercial or financial relationships that could be construed as a potential conflict of interest.

## Publisher's Note

All claims expressed in this article are solely those of the authors and do not necessarily represent those of their affiliated organizations, or those of the publisher, the editors and the reviewers. Any product that may be evaluated in this article, or claim that may be made by its manufacturer, is not guaranteed or endorsed by the publisher.

## References

[1] Bauman CA Barkema HW Dubuc J Keefe GP Kelton DF. Identifying management and disease priorities of Canadian dairy industry stakeholders. J Dairy Sci. (2016) 99:10194–203. 10.3168/jds.2016-11057 27720160

[2] Solano L Barkema HW Pajor EA Mason S LeBlanc SJ Zaffino Heyerhoff JC. Prevalence of lameness and associated risk factors in Canadian Holstein-Friesian cows housed in freestall barns. J Dairy Sci. (2015) 98:6978–91. 10.3168/jds.2015-9652 26254526

[3] Van Huyssteen M Barkema HW Mason S Orsel K. Association between lameness risk assessment and lameness and foot lesion prevalence on dairy farms in Alberta, Canada. J Dairy Sci. (2020) 103:11750–61. 10.3168/jds.2019-17819 32981721

[4] Bruijnis MRN Hogeveen H Stassen EN. Assessing economic consequences of foot disorders in dairy cattle using a dynamic stochastic simulation model. J Dairy Sci. (2010) 93:2419–32. 10.3168/jds.2009-2721 20494150

[5] Cramer G Lissemore KD Guard CL Leslie KE Kelton DF. The association between foot lesions and culling risk in Ontario Holstein cows. J Dairy Sci. (2009) 92:2572–9. 10.3168/jds.2008-1532 19447989

[6] Green LE Hedges VJ Schukken YH Blowey RW Packington AJ. The impact of clinical lameness on the milk yield of dairy cows. J Dairy Sci. (2002) 85:2250–6. 10.3168/jds.S0022-0302(02)74304-X 12362457

[7] Hernandez J Shearer JK Webb DW. Effect of lameness on the calving-to-conception interval in dairy cows. J Am Vet Med Assoc. (2001) 218:1611–4. 10.2460/javma.2001.218.1611 11393375

[8] Bruijnis M. Foot Disorders in Dairy Cattle: A Socio-Economic Approach to Improve Dairy Cow Welfare [Dissertation]. Wageningen University, Wageningen (2012).

[9] Van Nuffel A Zwertvaegher I Pluym L Van Weyenberg S Thorup MV Pastell M. Lameness detection in dairy cows: Part 1. How to distinguish between non-lame and lame cows based on differences in locomotion or behavior. Animals. (2015) 5:838–60. 10.3390/ani5030387 26479389 PMC4598709

[10] National Farm Animal Care Council. Canadian Code of Practice for the Care and Handling of Dairy Cattle. (2009).Available online at: https://www.nfacc.ca/pdfs/codes/dairy_code_of_practice.pdf (accessed July 22, 2019).

[11] Foditsch C Oikonomou G Machado VS Bicalho ML Ganda EK Lima SF. Lameness prevalence and risk factors in large dairy farms in upstate New York. Model development for the prediction of claw horn disruption lesions. PLoS One. (2016) 11:e0146718. 10.1371/journal.pone.0146718 26795970 PMC4721874

[12] Newsome R Green MJ Bell NJ Chagunda MGG Mason CS Rutland CS. Linking bone development on the caudal aspect of the distal phalanx with lameness during life. J Dairy Sci. (2016) 99:4512–25. 10.3168/jds.2015-10202 27060810

[13] Griffiths BE Grove White D Oikonomou G. A cross-sectional study into the prevalence of dairy cattle lameness and associated herd-level risk factors in England and Wales. Front Vet Sci. (2018) 5:65. 10.3389/fvets.2018.00065 29675419 PMC5895762

[14] Ritter C Adams CL Kelton DF Barkema HW. Factors associated with dairy farmers' satisfaction and preparedness to adopt recommendations after veterinary herd health visits. J Dairy Sci. (2019) 102:4280–93. 10.3168/jds.2018-15825 30852012

[15] Leach KA Whay HR Maggs CM Barker ZE Paul ES Bell AK. Working towards a reduction in cattle lameness: 1. Understanding barriers to lameness control on dairy farms. Res Vet Sci. (2010) 89:311–7. 10.1016/j.rvsc.2010.02.014 20363487

[16] Bruijnis M Hogeveen H Garforth C Stassen E. Dairy farmers' attitudes and intentions towards improving dairy cow foot health. Livestock Sci. (2013) 155:103–13. 10.1016/j.livsci.2013.04.005

[17] Adams CL Kurtz SM. Skills for Communicating in Veterinary Medicine, 1st Edn. Oxford: Otmoor Publishing; Parsippany, NJ: Dewpoint Publishing (2017).

[18] Ritter C Jansen J Roche S Kelton DF Adams CL Orsel K. Invited review: determinants of farmers' adoption of management-based strategies for infectious disease prevention and control. J Dairy Sci. (2017) 100:3329–47. 10.3168/jds.2016-11977 28237585

[19] Janz NK Becker MH. The health belief model: a decade later. Health Educ Q. (1984) 11:1–47. 10.1177/109019818401100101 6392204

[20] Ajzen I. The theory of planned behavior. Organ Behav Hum Decis Process. (1991) 50:179–211. 10.1016/0749-5978(91)90020-T

[21] Leung L. Validity, reliability, and generalizability in qualitative research. J Fam Med Primary Care. (2015) 4:324–7. 10.4103/2249-4863.161306 26288766 PMC4535087

[22] Vasileiou K Barnett J Thorpe S Young T. Characterising and justifying sample size sufficiency in interview-based studies: systematic analysis of qualitative health research over a 15-year period. BMC Med Res Methodol. (2018) 18:148. 10.1186/s12874-018-0594-7 30463515 PMC6249736

[23] Finfgeld-Connett D. Generalizability and transferability of meta-synthesis research findings. J Adv Nurs. (2010) 66:246–54. 10.1111/j.1365-2648.2009.05250.x 20423407

[24] Braun V Clarke V. Using thematic analysis in psychology. Qual Res Psychol. (2006) 3:77–101. 10.1191/1478088706qp063oa32100154

[25] Green J Thorogood N. Qualitative Methods for Health Research. 4th ed. London: Sage Publications Inc (2018). p. 440.

[26] Creswell JW. Qualitative Inquiry and Research Design: Choosing Among Five Approaches, 2nd Edn. Washington, DC: Sage Publications Inc.(2007).

[27] Brennan ML Wright N Wapenaar W Jarratt S Hobson-West P Richens IF. Exploring attitudes and beliefs towards implementing cattle disease prevention and control measures: a qualitative study with dairy farmers in Great Britain. Animals. (2016) 6:61. 10.3390/ani6100061 27727168 PMC5082307

[28] Saldana J. The Coding Manual for Qualitative Researchers, 2nd Edn. Thousand Oaks, CA: Sage Publications Inc.

[29] Harvey N Holmes CA. Nominal group technique: an effective method for obtaining group consensus. Int J Nurs Pract. (2012) 18:188–94. 10.1111/j.1440-172X.2012.02017.x 22435983

[30] Dutton-Regester KJ Wright JD Rabiee AR Barnes TS. Understanding dairy farmer intentions to make improvements to their management practices of foot lesions causing lameness in dairy cows. Prev Vet Med. (2019) 171:104767. 10.1016/j.prevetmed.2019.104767 31518830

[31] Cutler JHH Rushen J de Passillé AM Gibbons J Orsel K Pajor E. Producer estimates of prevalence and perceived importance of lameness in dairy herds with tiestalls, freestalls, and automated milking systems. J Dairy Sci. (2017) 100:9871–80. 10.3168/jds.2017-13008 28987585

[32] Jäger F. Social Psychological Concept of Normality: An Examination of the Emergence, Perpetuation and Shift of Unnoticed Standards, and Their Influence on Behavior and Social Dynamics [Dissertation]. Friedrich-Schiller-Universität Jena, Jena, Germany (2018).

[33] Artemiou E Adams CL Vallevand A Violato C Hecker KG. Measuring the effectiveness of small-group and web-based training methods in teaching clinical communication: a case comparison study. J Vet Med Educ. (2013) 40:242–51. 10.3138/jvme.0113-026R1 23975067

[34] Ritter C Adams CL Kelton DF Barkema HW. Clinical communication patterns of veterinary practitioners during dairy herd health and production management farm visits. J Dairy Sci. (2018) 101:10337–50. 10.3168/jds.2018-14741 30172401

