# Supplementary material for: Producer Perceptions Toward Prevention and Control of Lameness in Dairy Cows in Alberta Canada: A Thematic Analysis
Source: Front Vet Sci. 2022 Feb 8;9:812710. doi: 10.3389/fvets.2022.812710 (PMC8861376; doi:10.3389/fvets.2022.812710)
Supplement: Supplementary file 1 [file Data_Sheet_1.pdf]

Appendix

Interview guideline

UNIVERSITY OF CALGARY

ID: **Date:**

**** As with any qualitative interview guide, these questions are suggestions of what will be discussed in the interview and cover the main themes to discuss. ****

Start time:

Hello!
Thank you for agreeing to be interviewed. As you already know the purpose of this study is to gain thorough understanding of lameness control and prevention strategies implemented by dairy farmers and the perceived roadblocks in being successful.
As we discussed when going over the consent form, this interview is totally confidential, and the audio-recoding well be anonymized. I have an outline of questions I would like to ask you and I will be taking some notes during our discussion.
Please feel free to add any comments whenever you wish. Is there anything you would like to ask before we start?

Demographics

- Telling us about your farm
  - How long have you been farming?
  - What are your responsibilities on the farm?
  - For how long are you in this position with these responsibilities?
  - In which generation is the farm owned by your family?
  - How long have you had dairy cattle?
  - Which agricultural education do you have?

Attitude and Beliefs

- What poses the biggest threat on your farm?
- What comes to your mind when you think about lameness?
- On a scale from 1 (lowest) to 10 (highest), how important is lameness to you?
  - What is more important than lameness to you?
- What does lameness on a farm mean to you?
  - How likely is a farm to have lameness?

    - How do you feel when someone starts talking about prevention and control?
- Is taking actions to prevent or control lameness really worth it?
    - Why would you prevent or control lameness on your farm?
    - Have you already implemented prevention strategies and if how do they look like? If not, what led you to that decision.
- What do other people think you should be doing to prevent or control lameness on your farm?
    - Who are the people you think of when we say "preventions and controls"?
- Who has an opinion on what you should be doing? What is that opinion?
- What is your ideal measure for lameness prevention and control?
    - What would it do?
    - How would it be used?
- How do you decide which measures to implement on your farm?
    - How do you decide what to do on your farm?
    - What would make you implement a change directed at lameness prevention?
- Are lameness preventions and controls something you think you are really able to do?
    - Can you prevent or control lameness on your farm?
    - If you can prevent it, do you think that is the better strategy compared to react if you already have a case of lameness?

Knowledge

- In your opinion, what are the main causes of lameness?
    1. Where does this knowledge/information come from?
- If I say lameness preventions and controls, what does it make you think of?
    1. What do you picture?
- Do you feel confident about your knowledge about lameness?
    1. How does your knowledge contribute to your daily work routine?
- Can you tell me something on how you determine a lame cow?
- Did you receive any training in lameness diagnostic?
- How do you make decisions if a cow needs to be treated because of lameness?
- Do you have a strategy do prevent lameness on your farm and how does this look like?
- What do you think is effective in term of lameness prevention?

External stimuli (information sources)

- Would you describe your veterinarian as a trusted source of information on lameness?
    1. If your veterinarian is not your number one source, who else is considered an important source?
    2. What does their opinion of your ability to prevent and control lameness mean to you?
- What other people/organizations influence your decision-making on lameness prevention and control?
    1. How does the mandatory proAction assessment impact your decision-making?
- Do you think it is important to talk to other farmers about their prevention strategies in terms of lameness?
    1. If you talk to other farmers about that, do you have the feeling that they have good knowledge about lameness?
    2. Did it help you so far in mitigating lameness on your farm when implementing strategies other farmers talked about?
    3. What is the biggest roadblock, if there are any, other farmers talked to you about to mitigate lameness on their farms?

- What kind of support do you wish from the outside and from whom?

Concluding questions

- Is there anything you think I might have missed, or would like to add?
- How did being audiotaped influence you?

Finish time:

References:

Bruijnis, M., Hogeveen, H., Garforth, C., Stassen, E. 2013. Dairy farmers' attitudes and intentions towards improving dairy cow foot health. Livest. Sci. 155, 103-113.

Brennan, M.L., Wright, N., Wapenaar, W., Jarratt, S., Hobson-West, P., Richens, I.F., Kaler, J., Buchanan, H., Huxley, J.N., O'Connor, H.M. 2016. Exploring attitudes and beliefs towards implementing cattle disease prevention and control measures: a qualitative study with dairy farmers in Great Britain. Animals. 6, 61; doi:10.3390/ani6100061
